# Role and mechanism of lncRNA under magnetic nanoparticles in atrial autonomic nerve remodeling during radiofrequency ablation of recurrent atrial fibrillation

**DOI:** 10.1080/21655979.2021.2024324

**Published:** 2022-02-03

**Authors:** Weifeng Jiang, Ming Xu, Mu Qin, Daoliang Zhang, Shaohui Wu, Xu Liu, Yu Zhang

**Affiliations:** a Department of Cardiology, Shanghai Chest Hospital, Shanghai Jiao Tong University, Shanghai, China; b Department of Cardiology, The People’s Hospital of Suzhou New District, Suzhou City, China

**Keywords:** Atrial fibrillation, magnetic nanoparticles, CANR, lncRNA, high throughput sequencing

## Abstract

It aimed to investigate the mechanism of magnetic nanoparticles (MNPs) on atrial fibrillation and effect of n-isopropyl acrylamide coated MNPs (NIPA-co-MN) on the treatment of atrial fibrillation. Ten beagles weighing 20 − 25 kg were randomly divided into test group and control group. Dogs with atrial fibrillation were set as test group, and non-atrial fibrillation dogs as control group. The expression of long non-coding RNA (lncRNA) differentially expressed in the right anterior adipose pad in atrial fibrillation and non-atrial fibrillation dogs was detected by high-throughput sequencing. The relationship between lncRNA and cardiac autonomic nerve remodeling (CANR) was explored. In addition, 20 beagles weighing 20–25 kg were selected to study the therapeutic effect of n-isopropylacrylamide magnetic nanoparticles (NIPA-co-MN) on atrial fibrillation, and statistical analysis was performed. The volume and number of new neurons in the anterior right fat pad of atrium of test group were larger than the control group. The test group dogs produced 45 brand-new lncRNA, including 15 up-regulated transcripts and 30 down-regulated transcripts. MNPs injection can slow down the reduction of ventricular rate in right inferior ganglion plexus. The anterior right ganglion plexus resulted in a reduced amplitude of sinus tachyarrhythmia. This study provided references for the discovery of new diagnostic biomarkers or therapeutic targets and for the treatment of patients with atrial fibrillation.

## 1. Introduction

Cardiovascular disease is one of the most harmful diseases faced by the elderly and has become the leading cause of death in the world. Due to its high incidence and long course of disease, patients with cardiovascular diseases not only endure the physical discomfort caused by the illness, but also bear the high cost of treatment [1,2,3]. Atrial fibrillation is the disorder of the atria caused by many small reentry activities from atrium dominated the reentry loops. The overall incidence of atrial fibrillation is 0.4%, which increases with age, reaching 10% in people over 75 years old [4,5,6]. The therapeutic effects of atrial fibrillation remain unsatisfactory regardless of the development of drugs, surgery, and radiofrequency catheter ablation for atrial fibrillation. The lack of clarity of the pathogenesis of atrial fibrillation is a key constraint to the poor therapeutic effect of atrial fibrillation [7,8]. Although the condition of atrial fibrillation has been discovered, the relevant triggering mechanism of atrial fibrillation is not very clear. In recent years, the role of the autonomic nervous system in the occurrence of atrial fibrillation has attracted the attention of many scholars. The autonomic remodeling of the heart begins after atrial fibrillation or any other heart disease that can cause atrial fibrillation. There has been increasing evidence of abnormal autonomic nervous system, including sympathetic, parasympathetic neural networks, and intrinsic involvement in the pathogenesis of atrial fibrillation. Khan et al. (2019) [9] found that the sympathetic and parasympathetic nervous systems interacted with the cardiac ganglion plexus during the development of atrial fibrillation. Therefore, there was autonomic nerve dysfunction in atrial fibrillation. Selective ablation and other methods could reduce autonomic innervation and reduce the incidence of spontaneous or induced atrial arrhythmia. Yu et al. (2017) [10] analyzed the metabolite trimethylamine N-oxide produced by intestinal microorganisms, to understand the relationship between trimethylamine N-oxide and the autonomic nervous system. It was found that trimethylamine N-oxide could increase the electrophysiological instability of the atrial of normal dogs and aggravate the condition of atrial fibrillation in dogs by aggravating autonomic nerve remodeling.

The autonomic nervous system plays a role in the progression of atrial fibrillation. Although a new approach to targeted nerve ablation using magnetic nanoparticles is a technological advance, it should be viewed with caution and optimism. Based on the biodegradable nature of magnetic nanoparticles, the clinical use of a targeted therapeutic agent with potential good safety can greatly improve the therapeutic efficacy of patients with atrial fibrillation. However, there are several problems with the magnetic nanoparticle approach. In view of the interconnectedness of the atrial ganglion plexus, it is necessary to further describe the hierarchical structure of the atrial ganglion plexus and the correlation between the minimum and specific number of the atrial ganglion plexus and atrial fibrillation, because single or multiple-ganglion plexus can’t cause atrial fibrillation [11].

Therefore, the lncRNA involved in the CANR in atrial fibrillation was analyzed in this study. The treatment of atrial fibrillation via magnetic nanoparticles (MNPs) was studied, which was expected to provide certain theoretical basis for the clinical diagnosis and treatment of atrial fibrillation.

## 2. Materials and methods

### 2.1 Study objects and grouping

To ensure no interference from other conditions, the relationship between lncRNA and CANR in atrial fibrillation and the effect of magnetic nanoparticles for the treatment of atrial fibrillation were analyzed using different experimental objects.

To explore the relationship between lncRNA and atrial fibrillation CANR, 10 beagles (purchased from the Experimental Animal Center of Shandong University) of 20–25 kg were selected, regardless of gender. They were randomly divided into the test group and the control group, with five dogs in each group. Pacemaker electrodes were placed on the right auricle of the heart of all dogs, while the control group dogs were only placed without pacemaker, and fed for 4 weeks for observation. In the test group, the atrial fibrillation dog model was established with pacemakers of 400 times/min for 4 weeks. To detect atrial fibrillation, surface electrocardiogram was performed at least once a week in both groups. Tissue was extracted from the anterior right fat pad of atrium of the control group and the test group for immunohistochemistry staining of nerve cells with Protein Gene Product 9.5. The neural density was calculated to verify the occurrence of atrial fibrillation mediated neural remodeling.

To analyze the effect of magnetic nanoparticles in the treatment of atrial fibrillation, 20 beagles weighing 20–25 kg were selected, regardless of gender. The dogs were anesthetized with pentobarbital sodium and ventilated under positive pressure with the centrosomes kept at about 37°C.

### 2.2 Animal preparation

Magnetic nanoparticles were utilized to induce atrial fibrillation in animals by stimulating the anterior right-ganglion plexus or the inferior right-ganglion plexus. The induced minimum voltage-level value served as the threshold value. Six animals were randomly selected, and 0.5 mL magnetic nanoparticles containing 0.4 mg NIPA-co-MN were injected into the anterior right-ganglion plexus of the animals. The maximum value of sinus tachycardia induced by stimulating the anterior right-ganglion plexus without inducing atrial fibrillation was measured at 1 h, 2 h, and 3 h after injection. Moreover, another four animals were randomly selected, and a columnar permanent magnet (2600 G, surface area 2 cm^2^) was sutured to the anterior right fat pad of atrium on the epicardial surface containing the inferior right ganglion plexus to capture magnetic nanoparticles directionally. The trocar was inserted into the circumflex branch of the coronary artery, and 1 mL magnetic nanoparticles were injected into the circumflex branch of the cyclotronic artery within 1 minute. Function of the anterior right ganglion plexus and the inferior right ganglion plexus was evaluated at 1 h, 2 h, and 3 h as described above [12].

### 2.3 MNPs preparation

The core of MNPs is the magnet and Fe_3_O_4_, which is formed by the precipitation of ferrous and ferric salts in the presence of the basic solution and the surface activator multi-sodium salt. In this process, the magnetic core binds to the poly-hydrogel matrix (shell) to provide strong adsorption, which prevents the magnetic nanopore core from dispersing from the poly-shell. Moreover, capsules that treat loaded NIPA-co-MN were also fabricated. The temperature was set at about 37°C, which was the same as the human body, for the drug release would be enhanced at body temperature [13].

### 2.4 Immunohistochemical staining

The cleaned sections were placed on the 2.3-amino propyltriethoxy silane (ZLI-9001), diluted with 1:50 acetone. After 20–30 seconds, they were taken out, then rinsed the unbonded 2.3-amino propyltriethoxy silane into pure acetone solution or distilled water, and dried in the fume hood. When this section was retrieved, the tissues were in place at one step, and the presence of bubbles were minimized, so as not to affect the staining results. The cleaned sections were put into the solution where histogrip (Shanghai Yisheng Biotechnology Co., LTD.) was diluted by 1:50 ratio of acetone (ZLI-9003) and left for 1 ~ 2 minutes. Then, they were quickly cleaned with double steam water for three times, dried at 25°C or baked in an oven at 60°C for one hour. Next, they were put in a box for later use. The clean and dry sections were placed in the polylysine solution diluted in 1:10 deionized water and soaked for 5 minutes. Baked in the oven at 60°C for one hour or overnight at 25°C, they were packed in boxes for later use. The appliances used in the test were all non-glass products.

Paraffin sections were dewaxed into water and incubated with 3% H_2_O_2_ at 25°C for 5–10 minutes to eliminate the activity of endogenous peroxidase, and rinsed with distilled water and soaked in PBS for 5 minutes. Normal goat serum (diluted with PBS) was sealed and incubated for 10 min at 25°C. The serum was poured without washing, and the primary antibody or primary antibody working solution was added in appropriate proportion, and incubated at 37°C for 1 ~ 2 hours or 4°C overnight. The sections were rinsed with PBS, 5 min ×3 times, and Biotin-labeled secondary antibody (1%BSA-PBS dilution) was added and incubated at 37°C for 10 ~ 30 minutes. Alternatively, the second-generation biotin-labeled secondary antibody working solution was added by drops and incubated at 37°C or 25°C for 10 ~ 30 minutes. The sections were rinsed with PBS, 5 min ×3 times. Drops of horseradish enzyme labeled streptomycin (diluted with PBS) were incubated at 37°C for 10 ~ 30 minutes. The sections were rinsed with PBS, 5 min ×3 times, and DAB color developing agent (Beijing Solar bio-technology Co., LTD.) was adopted. Tap water was used to fully rinse them, and the sections were re-dyed and sealed [14].

### 2.5 Determination of nerve density

The computer aided image analysis system was used to measure the nerve density according to the result of the staining. The computer can distinguish between positive staining results and calculate the corresponding area of positive staining results. The ratio of the calculated area to the total area represented the nerve density, which magnified each section 20 times for observation. Three fields with the highest nerve density were reserved for statistical analysis, and the average density value of the whole slice was the mean of the nerve density of the three fields [15].

### 2.6 Sample RNA extraction

The extraction steps of sample RNA were as follows. The lysed cells were cryopreserved and left for 5 min at 25°C for complete dissolution. The cells were then cleaved and separated. 0.2 mL chloroform was added to each 1 mL sample cracked by Trizol reagent, and the tube cover was tightly closed. The tube was incubated for 2 to 3 minutes at 15°C to 30°C after 15 seconds of manual vigorous shaking, and centrifuged at 12,000rpm for 15 min at 4°C. After centrifugation, the mixture would be divided into the lower layer of the red phenol chloroform phase, the middle layer, and the colorless aqueous phase of the upper layer. The RNA was all distributed in the aqueous phase. The volume of the aqueous phase upper layer was approximately 60% of the Trizol reagent added to the homogenizer (Tiangen Biotech Technology (Beijing) Co., LTD.). The upper layer of the aqueous phase was transferred to a clean RNA-free centrifuge tube. Isopropyl alcohol was added to precipitate the RNA. After mixing, the RNA was incubated at 15°C to 30°C for 10 minutes, and centrifuged at 12,000rpm at 4°C for 10 minutes. At this point, the RNA precipitation that was not visible before centrifugation would form colloidal precipitation blocks on the bottom and side walls of the tube. The supernatant was removed, added with at least 1 mL 75% ethanol (75% ethanol prepared with DEPC (diethyl pyrocarbonate) water) to each sample lysed with 1 mL Trizol reagent, and the RNA precipitation was cleaned. After mixing, the samples were centrifuged at 7,000rpm at 4°C for 5 minutes. Most ethanol solution was carefully absorbed and the RNA precipitation was allowed to dry in air at 25°C for 5–10 minutes. Finally, the RNA precipitate was dissolved, 40 μL RNA-free water should be added and it was blown for several times with a gun to completely dissolve it. The obtained RNA solution was stored at −80°C for use [16].

### 2.7 High throughput second generation sequencing

After total RNA was extracted by the Trizol method, high-throughput lncRNA sequencing was performed on two mixed samples using *Illumine Hiseq 2500* sequencing platform (Illumina, Inc., San Diego) via a single-end 50-bp sequencing method. According to the characteristics of *Illumine Hiseq 2500* sequencing data with low quality fractions concentrated at the end, *Trim Galore* was used to dynamically remove the low-quality fragments from the 3ʹ end of the sequencing data and connect the sequence fragments. *FastQC* was used to analyze the quality of pretreated data, nucleic acid composition preference, and GC content control. Then, for each sample, *Tophat* was used to compare the pretreated sequence with the reference genome sequence. First, the whole sequence was compared with the known transcript sequence, and then the segmented sequence was compared to the genome. Next, *Reads* repeatability assessment, reference genome density distribution assessment, *Reads* comparison distribution assessment, transcript coverage uniformity distribution assessment, variable shear site annotation, and variable shear saturation assessment were performed for the *Reads* comparison genome results. The next step was transcript assembly and prediction of new transcripts. The above comparison results were assembled using *cuffLinks* based on the location of known transcripts on the genome. Finally, FragmentsPer Kilobase per Million (FPKM) was used to calculate the transcription expression. *Cuffdi* was used to screen known transcripts and new long-chain non-coding transcripts that were differentially expressed between the two groups. Transcripts with *P* ≤ 0.05 and/or ≥2 times differential expression range were defined as differential expression transcripts [17].

### 2.8 cDNA synthesis and real-time fluorescence quantitative PCR sample detection

cDNA synthesis: all cDNA samples were configured with real-time fluorescence quantitative PCR reaction system. The solution was mixed and centrifuged at 6,000rpm for a short time. Before reverse transcriptase was added, the mixture was bathed in a dry bath at 70°C for 3 minutes. Immediately after removal, the mixture was bathed in ice water until the temperature inside and outside the tube became the same. Then, the mixture was added with 0.5 μL reverse transcriptase and bathed at 37°C for 60 minutes. After being taken out, the reverse transcription final solution was obtained in dry bath at 95°C for 3 minutes and was stored at −80°C for use. Real-time fluorescence quantitative PCR refers to a method that adds fluorescent groups to the PCR reaction system, adopts fluorescence signal accumulation to monitor the whole PCR process in real time, and finally conducts quantitative analysis of the unknown template through the standard curve [18].

qRT-PCR sample detection: all cDNA samples were configured with real-time fluorescence quantitative PCR reaction system. The obtained real-time fluorescence quantitative PCR reaction system was quickly centrifuged at 6,000rpm. The prepared PCR reaction solution was placed on a real-time fluorescence quantitative PCR instrument (Applied Biosystems) for PCR amplification. The reaction conditions were as follows: pre-denaturation was conducted at 93°C for 2 minutes, followed by 40 cycles at 93°C for 1 minute, 55°C for 1 minute, and 72°C for 1 minute, and then gene amplification was conducted at 72°C for 7 minutes [19].

### 2.9 Statistical methods

SPSS 26.0 was used for data processing. Continuous variables were expressed as mean plus or minus standard deviation. ($\overset{ˉ}{x}$±s) One-way analysis of variance was used for inter-group comparison. All *P* values were double-tailed test, where *P* < 0.05 indicated statistically significant differences, and *P* < 0.01 indicated statistically significant differences.

## 3. Results

Beagle dogs were used as the research object to build atrial fibrillation model, and high-throughput sequencing method was adopted to conduct lncRNA sequencing and detect differential expression. The therapeutic effect and mechanism of magnetic nanoparticles loaded with NIPA-co-MN on atrial fibrillation were analyzed by MNPs microinjection.

### 3.1 Occurrence of autonomic remodeling in atrial fibrillation dogs

**Figure 1.**
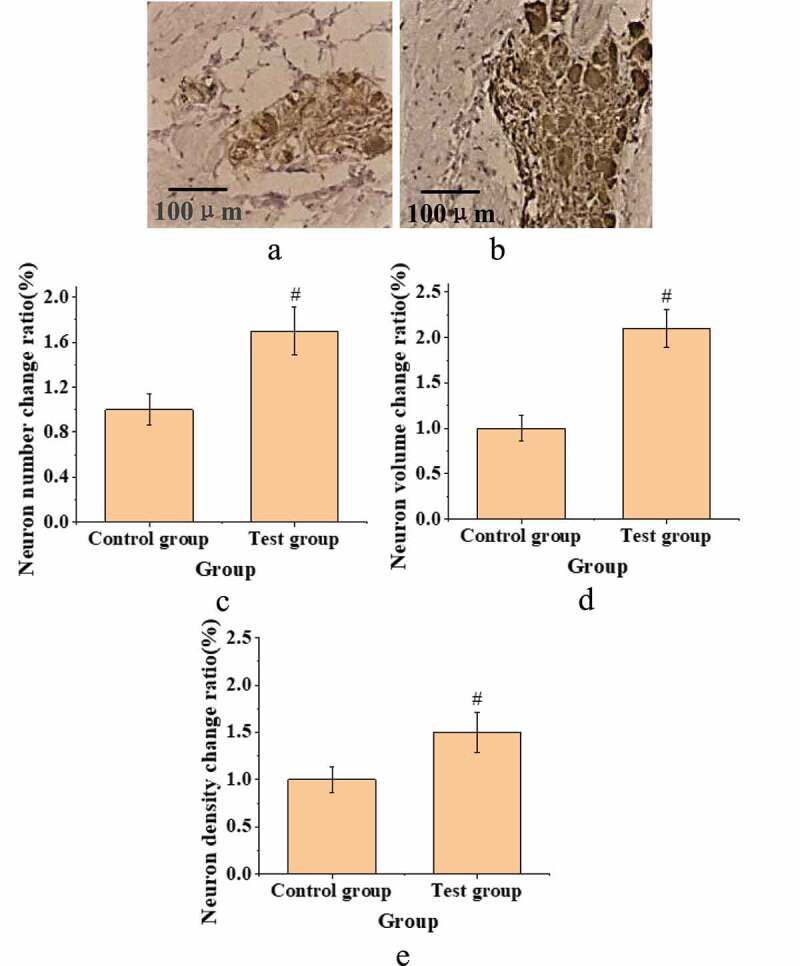
The status of new-born neurons in the anterior right fat pad of atrium in both groups. (A: immunohistochemical staining of control group; B: immunohistochemical staining of the test group; C. comparison diagram of the number of neurons in each group; D. comparison of neuron volume changes in each group; E. comparison of neuron density changes in each group (#: compared with control group, *P* < 0.05).

The atrial fibrillation model was successfully established in all animals in the test group through the 4 weeks of pacing mentioned in the methods. Immunohistochemistry staining and neuro-densitometry were implemented to verify the occurrence of neural remodeling in the anterior right fat pad of atrium. The number of PGP9.5 positive fibers in the myocardial space of the test group was significantly increased compared with the control group. In addition, the volume and number of new neurons in the anterior right fat pad of atrium in the test group were larger (Figure 1). Moreover, the nerve density of the test group was significantly increased relative to that of the control group (*P* < 0.05).

### 3.2 lncRNA differential expression results

Cuffcompare was adopted to compare the collation results of Cufflinks with the reference annotations, 2,593 transcripts were obtained, and the transcripts contain pairs of exons. Cuffdiff was used to quantify the expression of the predicted new transcripts and known lncRNA, and then the difference lncRNA between samples was analyzed. A total of 558 lncRNAs with no less than 2 times expression difference (*P* < 0.05) were selected. Of which, 156 transcripts were down-regulated and 402 transcripts were up-regulated. Among the 558 lncRNAs, there were 45 newly discovered lncRNAs, including 15 up-regulated transcripts and 30 down-regulated transcripts (Table 1).

**Table 1.** Novel lncRNAs differentially expressed in the test group and the control group

lncRNA	Multiple	P value	lncRNA	Multiple	P value
lncRNA-052951	2.219	0.00301	lncRNA-042368	2.199	0.0339
lncRNA-108051	2.694	0.00005	lncRNA-060304	2.097	0.0347
lncRNA-019343	−2.331	0.0039	lncRNA-095627	−2.279	0.0109
lncRNA-084333	2.319	0.0204	lncRNA-066916	2.711	0.0436
lncRNA-007769	−9.994	0.0207	lncRNA-023348	−10.962	0.0376
lncRNA-002552	−15.493	0.0301	lncRNA-031978	−3.795	0.0005
lncRNA-100354	−2.012	0.0361	lncRNA-104781	5.219	0.0011
lncRNA-031251	−9.392	0.0017	lncRNA-113768	3.459	0.0006
lncRNA-064998	4.091	0.0311	lncRNA-067113	8.591	0.0019
lncRNA-112654	4.017	0.0005	lncRNA-096802	3.802	0.0013
lncRNA-079815	−2.569	0.01342	lncRNA-002212	−3.457	0.0413
lncRNA-065026	−2.344	0.02795	lncRNA-112101	4.302	0.00218
lncRNA-019960	−2.413	0.00165	lncRNA-011581	−13.012	0.02599
lncRNA-002213	−2.978	0.0143	lncRNA-008295	3.013	0.03577
lncRNA-036711	−2.131	0.0353	lncRNA-019687	−4.369	0.0091
lncRNA-065551	−3.564	0.0245	lncRNA-017223	2.796	0.01315
lncRNA-048371	−4.332	0.0011	lncRNA-008480	−12.407	0.0044
lncRNA-081339	−3.501	0.0314	lncRNA-050774	−25.319	0.0011
lncRNA-000416	−2.786	0.0175	lncRNA-032546	−2.379	0.00307
lncRNA-058834	−12.541	0.0094	lncRNA-026102	−2.337	0.04087
lncRNA-051148	−4.899	0.00031	lncRNA-011929	−3.479	0.002
lncRNA-085926	−14.321	0.04089			

### 3.3 Verification of sequencing results

**Figure 2.**
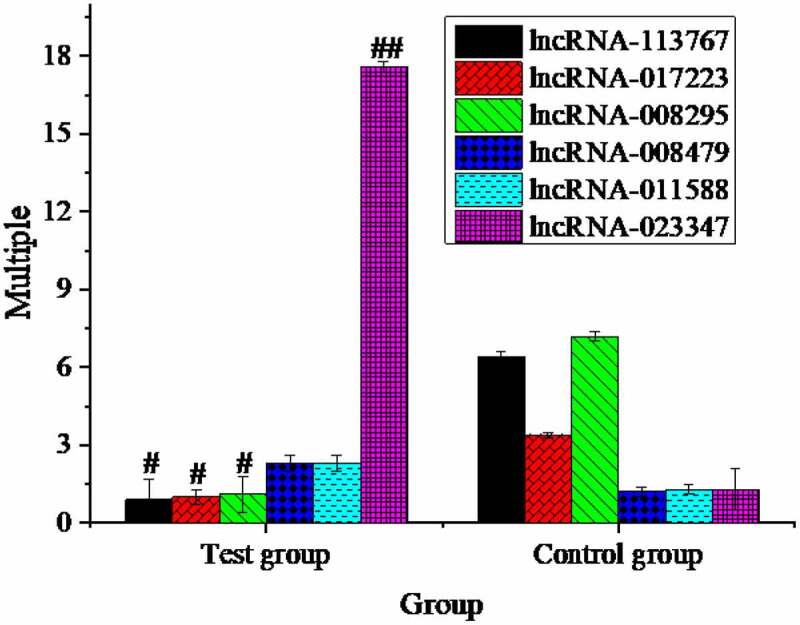
Expression multiple changes of lncRNA (#: compared with control group, *P* < 0.05; ##: compared with control group, *P* < 0.01.).

Six new transcripts were randomly selected for sequencing verification, including three up-regulated and three down-regulated new transcripts. The detection results showed that the expression trend of lncRNA was consistent with the detection results (Figure 2).

Except for lncRNA-008479 and lncRNA-011588, the differences between the other lncRNA groups and the control group were substantial. Although the differences in lncRNA-008479 and lncRNA-011588 weren’t considerable between the two groups, the basic decline trend was indicated.

### 3.4 MNPs microinjection results

**Figure 3.**
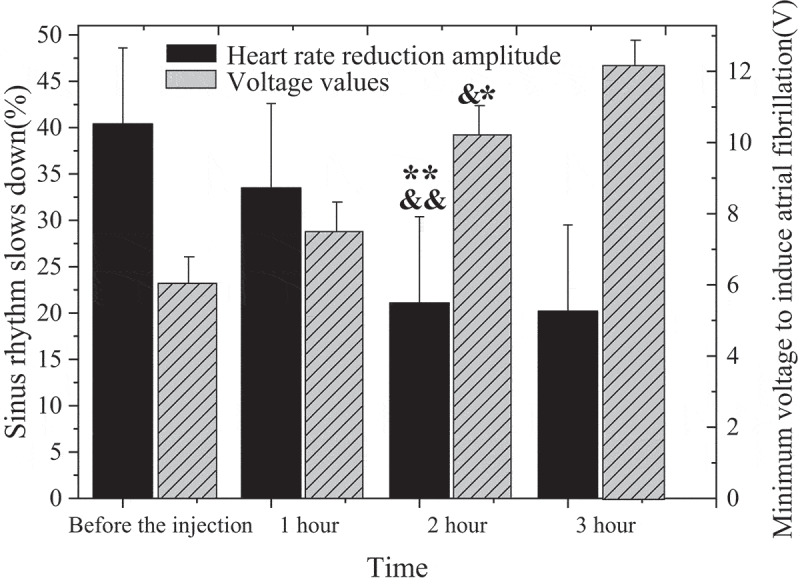
MNPs microinjection results (&: compared with that before injection, *P* < 0.05; &&: compared with that before injection, *P* < 0.01; *: compared with 1 h after injection, *P* < 0.05; **: compared with 1 h after injection, *P* < 0.01.).

After the anterior right-ganglion plexus of the animal was injected with 0.5 mL magnetic nanoparticles containing 0.4 mg NIPA-co-MN, the changes of sinus rhythm and minimum voltage were analyzed before and at 1, 2, and 3 h of NIPA-co-MN injection. Moreover, the maximum sinusoidal heart rate reduction caused by stimulating the anterior right ganglion plexus without inducing atrial fibrillation was measured. Sinus rhythm deceleration was described in Figure 3 in the form of a percentage.

In Figure 3, after stimulation of right anterior ganglion plexus in mice, sinus rhythm was slowed down by 40.4%±8.2% after injection of magnetic nanoparticles. However, the stimulation of the anterior right-ganglion plexus led to a decrease in the magnitude of sinus tachycardia after the injection. Sinus tachycardia (33.5%±9.1%) was induced by injection of magnetic nanoparticles for 1 h after stimulation of right anterior ganglion plexus. When the magnetic nanoparticles were injected for 2 h, the value decreased to 21.1%±9.3%, and the difference between groups was considerable (*P* < 0.01). Sinus rhythm decreased by 20.2%±9.3% after 3 h injection of magnetic nanoparticles. However, the difference between 3 h and 2 h injected magnetic nanoparticles were not considerable (*P* > 0.05).

The minimum voltage value of stimulating the anterior right-ganglion plexus to induce atrial fibrillation in animals was 5.9 ± 0.8 V before the magnetic nanoparticles were injected into animals. However, after the injection, the minimum voltage showed a trend of gradual increase. The voltage reached 7.5 ± 0.9 V after injection for 1 h. The voltage reached 10.2 ± 0.9 V after injection for 2 h, and the difference between groups was remarkable (*P* < 0.05). However, the voltage value reached 12.3 ± 0.9 V after the magnetic nanoparticles were injected for 3 h, but the difference between 3 h and 2 h of injection was not great (*P* > 0.05).

### 3.5 Results of MNPs injection through coronary artery

**Figure 4.**
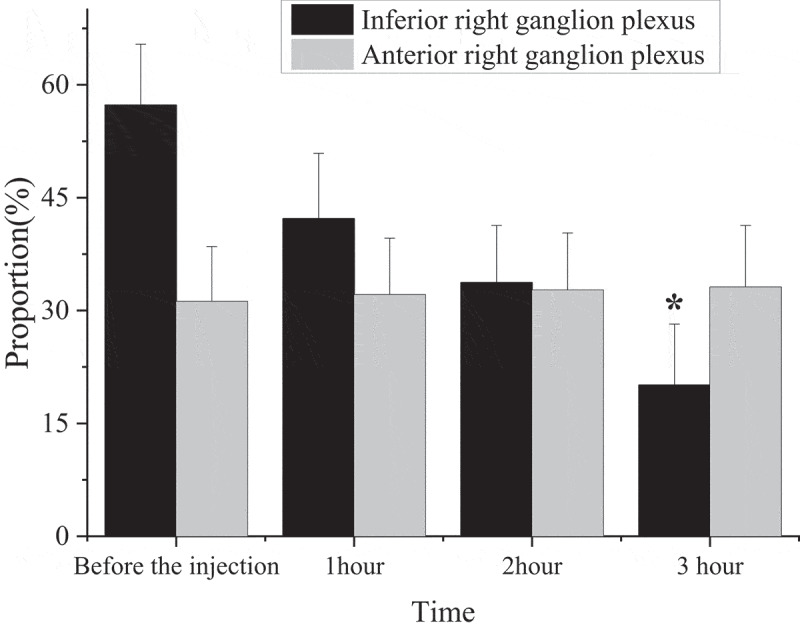
Functions of anterior right ganglion plexus and inferior right ganglion plexus (*: compared with injection for 2 h, *P* < 0.05.).

The functions of the anterior right-ganglion plexus and the inferior right-ganglion plexus of the animals were measured before and after the injection of magnetic nanoparticles into the meridians (Figure 4).

In Figure 4, the inferior right-ganglion plexus could slow down the ventricular rate of 57.3%±8.1% before the injection, which showed a downward trend with the injection time. At 1 h of injection, the inferior right-ganglion plexus reduced the magnitude of ventricular rate to 42.2%±8.7%. However, the value at 2 h after the injection was basically unchanged from that of 1 h. At 2 h of injection, the magnitude of the inferior right-ganglion plexus reduction in ventricular rate was reduced to 33.7%±7.6%. At 3 h of injection, the amplitude of inferior right-ganglion plexus decreased to 20.1 ± 8.1%, and the difference between 2 h and 3 h before the injection of magnetic nanoparticles was substantial (*P* < 0.05). The effect of the anterior right-ganglion plexus on decreasing sinus heart rate was not changed during the injection, which was 31.2%±7.3% before the injection and 33.1%±8.2% at 3 h, and the difference was not substantial.

**Figure 5.**
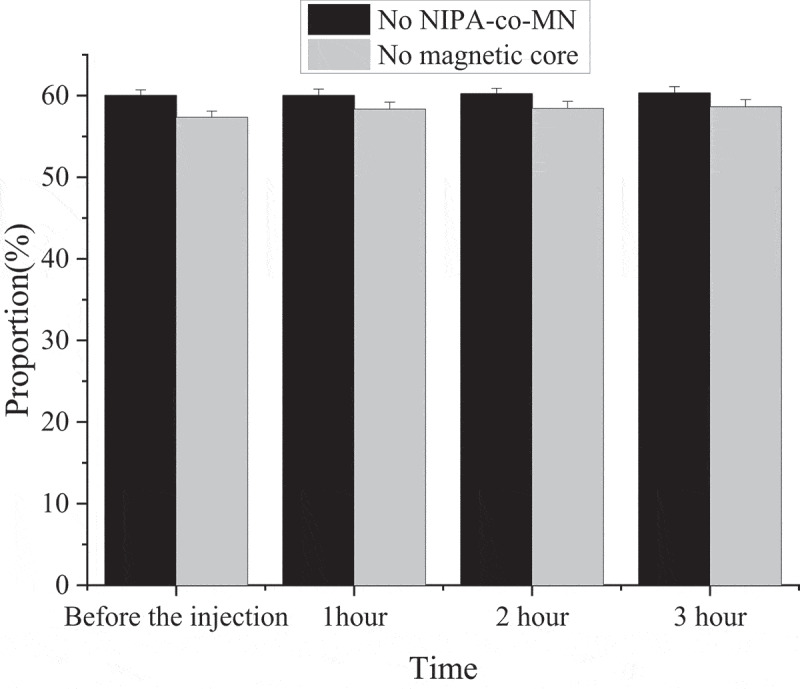
Injection effect of magnetic nanoparticles without NIPA-co-MN load or without magnetic cores.

The effect of the injection of magnetic nanoparticles without NIPA-co-MN load or without magnetic cores are shown in Figure 5.

In Figure 5, the functions of the inferior right-ganglion plexus didn’t change within 3 hours.

**Figure 6.**
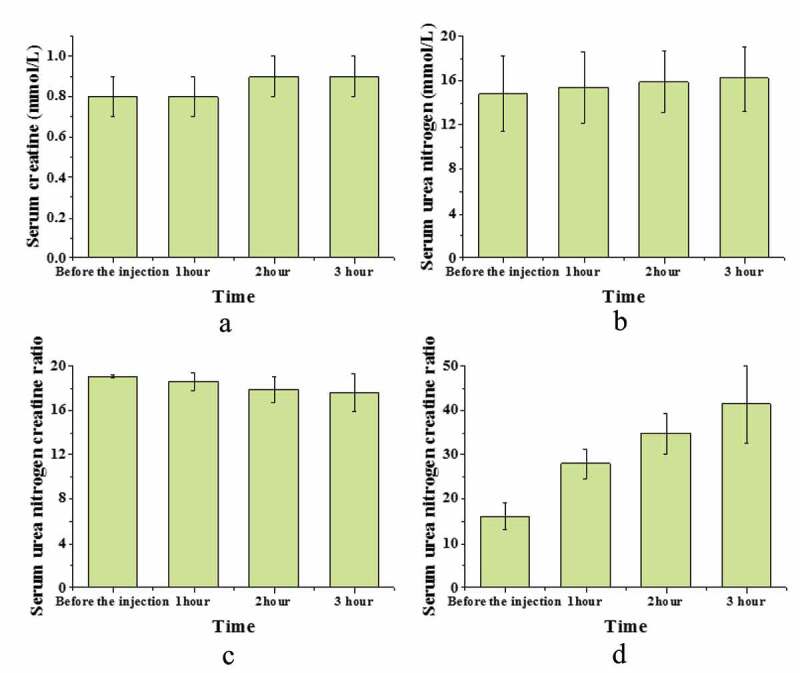
The indicators of NIPA-co-MN ventricular direct injection animals.

Except for alanine aminotransferase, other indicators showed no obvious changes, and alanine aminotransferase showed an increasing trend, but the difference between the groups was not substantial (*P* > 0.05) (Figure 6).

## 4. Discussion

There has been some progress in all types of heart disease and breakthroughs in the treatment of patients with arrhythmias. However, for patients with atrial fibrillation, recovery remains a huge challenge [20,21]. Currently, the commonly used drugs for the treatment of atrial fibrillation in the market generally have a series of problems, such as low recovery rate, large side effects, so the treatment of atrial fibrillation is in urgent need of new methods [22]. To treat atrial fibrillation, it is essential to understand its pathogenesis. Therefore, scholars conducted researches on the pathogenesis of atrial fibrillation [23,24]. It was generally believed that the activity of the cardiac autonomic nervous system played a role in the occurrence of atrial fibrillation. Therefore, the lncRNA involved in the CANR in atrial fibrillation was analyzed, and the application of magnetic nanoparticles in the treatment of atrial fibrillation was further studied.

Studies suggested that lncRNA of right anterior ganglion plexus in dogs with atrial fibrillation had certain unique expression profile. Forty-five differentially expressed transcripts were entirely new and unexplained lncRNAs. These results suggested that atrial fibrillation was related to CANR. This also indicated that lncRNA was involved in key nodes of CANR regulatory network. Qian et al. (2019) [25] constructed an atrial fibrillation associated lncRNA-mRNA network based on endogenous competitive RNA theory, and lncRNA-regulated interaction was found to be closely associated with the pathogenesis of atrial fibrillation by integrating probes to re-annotate the pipeline and microRNA-target regulation. The results obtained in this study were basically consistent with the above studies, indicating that the occurrence of atrial fibrillation was closely related to lncRNA.

Yu et al. (2018) [26] studied the process of encapsulating magnetic nanoparticles to treat atrial fibrillation, and found that this method can be used to treat arrhythmias associated with active cardiac autonomic nervous system in the early stage of atrial fibrillation, with less myocardial damage. O’Quinn et al. (2019) [27] studied the function of time-released nano-CaCl_2_ magnetic nanoparticles in regulating autonomic nerves and inhibiting postoperative atrial fibrillation. It was found that the injection of nano-CaCl_2_ into the left atrial ganglion plexus can induce apoptosis of nerve cells, regulate autonomic nerve function, and inhibit postoperative atrial fibrillation. Meanwhile, neuropathological studies indicated that acrylamide monomer and its analogues, including NIPA, were related to the toxic effects of inhibiting glycolytic enzymes (such as enolase) on neurons and axons [28]. In this study, magnetic nanoparticles containing NIPA-co-MN were injected into the vein to target the right lower ganglion plexus. The subsequent release of NIPA-co-MN may be used to reduce the activity of ganglion cells, and at the same time, it can reduce the amplitude of the right lower ganglion plexus and increase the minimum voltage value. treatment effect. This was consistent with the study of Yu et al. Therefore, the injection of magnetic nanoparticles containing NIPA-co-MN into the relevant active cardiac autonomic nervous system was a small invasive and low-cost drug-resistant atrial fibrillation treatment method.

## 5. Conclusion

Through the analysis of the pathogenesis and treatment of atrial fibrillation, it was found that magnetic nanoparticles containing NIPA-co-MN could affect the activity of ganglion cells and increase the minimum voltage value of atrial fibrillation by stimulating the right anterior ganglion plexus. In addition, the occurrence of atrial fibrillation was related to lncRNA, which can make the expression of lncRNA-008479 and lncRNA-011588 show a downward trend. Therefore, studies on lncRNA are expected to provide a new explanation for the CANR mechanism of atrial fibrillation and a new intervention target for the prevention and treatment of atrial fibrillation. Although some achievements are made, there are still some shortcomings. First, the sample size was small. As a result, the results may be affected by some accidental factors, increasing the feasibility and limitations of the results. Second, the experimental design does not fully consider the stimulus factors, and the medication time is too short. It is expected that this experiment will be further refined and studied in detail when conditions permit. Therefore, in the future study, the study results will be more reliable by increasing the number of samples and the duration of medication involved in the experimental process, to provide a basis for the treatment of central room fibrillation as soon as possible.

## Data Availability

All data, models, and code generated or used during the study appear in the submitted.
